# Editorial: The intersection of artificial intelligence and brain for high-performance neuroprosthesis and cyborg systems

**DOI:** 10.3389/fnins.2023.1133002

**Published:** 2023-02-24

**Authors:** Yu Qi, Yu Sun, Quanying Liu, Qiaosheng Zhang, Hanshu Cai, Qian Zheng

**Affiliations:** ^1^Affiliated Mental Health Center and Hangzhou Seventh People's Hospital, MOE Frontier Science Center for Brain Science and Brain-Machine Integration, Zhejiang University School of Medicine, Hangzhou, China; ^2^Key Laboratory for Biomedical Engineering of MOE of China, College of Biomedical Engineering and Instrument Science, State Key Laboratory of Brain-Machine Intelligence, Zhejiang University, Hangzhou, China; ^3^Department of Biomedical Engineering, Southern University of Science and Technology, Shenzhen, China; ^4^Department of Anesthesiology, New York University School of Medicine, New York City, NY, United States; ^5^Gansu Provincial Key Laboratory of Wearable Computing, School of Information Science and Engineering, Lanzhou University, Lanzhou, China; ^6^The State Key Lab of Brain-Machine Intelligence, Zhejiang University, Hangzhou, China

**Keywords:** brain-computer interface, artificial intelligence, brain signal analyses, machine learning, brain-like computing

Brain-computer interfaces (BCI) can build a novel pathway from the brain to external devices. Advanced developments of BCIs have enabled direct brain control of devices such as computer cursors or robotic arms and constructed neuroprosthesis that converts brain signals into speech and handwriting, which demonstrate great potential in neural rehabilitation (Pan et al., 2018; Anumanchipalli et al., 2019; Willett et al., 2021). However, current BCI systems still face critical challenges regarding accuracy, robustness, stability, and reliability in online control, which seriously hinders the clinical application of BCIs.

The intersection of artificial intelligence and the brain is promising to bring new developments in BCI systems. Machine learning technologies, especially deep neural networks, have demonstrated effectiveness in brain signal processing and analysis, and improved the performance of BCI systems in both accuracy and stability. The study Su et al. showed that a 3D convolutional neural network model with multiscale convolutional kernels could effectively improve emotion recognition performance with EEG signals compared with traditional approaches. The study Dai et al. proposed a novel feature selection method for high-dimensional biomedical data to improve both efficiency and accuracy in biomedical signal processing.

Artificial intelligence technology also shows the ability to help BCIs break through the current bottleneck problems. One critical challenge in practical BCI systems lies in that users have to recalibrate the BCI with repetitive training trials every day, which can be tedious and harms the user experience. The study Liu et al. demonstrated that the proposed transfer learning-based approach could maintain stable performance within-subject cross-day even with zero training, which could improve the BCI performance and reduce the calibration burden, facilitating practical applications of BCI systems.

The convergence of artificial intelligence and the brain also emerges novel forms of BCI systems. The study Lu et al. built a video target detection system with the brain-in-the-loop, by detecting video-induced P3 signals when the subjects inspect the video. With the strength of a graph convolutional network, high recognition performance was achieved. The study Du et al. constructed a BCI-based hand rehabilitation system with tactile-enhanced multi-sensory feedback and demonstrated that the vibrotactile enhancement had a reinforcing effect on sensorimotor brain activities and could promote the clinical application of BCIs in the rehabilitation of stroke patients.

Another novel and inspiring perspective toward the intersection of artificial intelligence and the brain is to use brain-like computing models to analyze brains. Brain-like computing models such as spiking neural networks have great potential in the effective representation and computation of brain signals with the natural biological plausible property. The study Singanamalla et al. proposed to learn effective EEG representations with a spiking neural network, and the latent spike representations reflected EEG features and improved the BCI performance with significance. The perspective can be inspiring for the field of neural signal processing.

Therefore, bringing together artificial intelligence and brain intelligence is promising to emerge novel technologies in BCIs from various perspectives. Artificial intelligence technologies can enable high-performance BCIs with accurate and stable control, and facilitate practical applications of BCIs and Neuroprosthesis.

## Author contributions

YQ wrote the editorial for the topic. All authors contributed to the article and approved the submitted version.
